# Partial Portacaval Shunt: Narrow Diameter H-Graft

**DOI:** 10.1155/1994/75457

**Published:** 1994

**Authors:** J. A. Myburgh

**Affiliations:** University of the Witwatersrand Medical School, York Road, Parktown Johannesburg 2193 South Africa


*HPB Surgery*, 1994, Vol. 8, pp. 57–76
Reprints available directly from the publisher
Photocopying permitted by license only

© 1994 Harwood Academic Publishers GmbH
Printed in Malaysia

### HPB INTERNATIONAL

**EDITORIAL & ABSTRACTING SERVICE**

JOHN TERBLANCHE, EDITOR
Department of Surgery
Medical School · Observatory 7925
Cape Town · South Africa
Telephone: (021) 406-6232 Ext. 2
Telefax (021) 448-6461

### PARTIAL PORTACAVAL SHUNT: NARROW DIAMETER H-GRAFT

### ABSTRACT

*Adam, R., Diamond, T. and Bismuth, H. (1992) Partial portacaval shunt: Renaissance of an old concept. Surgery, 111, 610–616.*

***Background.*** **Partial diversion of the portal system aims to redeuce portal pressure sufficiently to prevent variceal hemorrhage but still maintain adwquate hepatic portal flow.**
***Methods.*** **Partial portacaval shunts were performed in 25 patients with cirrhosis with portal hypertension and esophageal varices, either as a primary procedure ($n=16$) or for failure of endoscopic sclerotherapy ($n=9$), with ringed polytetrafluoroethylene prostheses (8, 10, or 12 mm).**
***Results.*** **All patients have now been followed up for at least 1 year. The operative mortality rate (2 months) was 4%. In 24 patients who survived beyond the initial perioperative period, there was no recurrence of variceal bleeding. Cumulative shunt patency (up to 4 years) is 96%. Acute encephalopathy was detected in two patients (8%), but no patients had signs of chronic encephalopathy. Intraoperative pressure measurements revealed a signficant correlation between decreasing diameter of the graft and the percentage reduction of the portacaval pressure gradient. Selective angiography, performed 1 year after surgery, revealed that hepatopetal flow was maintained in 70% of patients with a 10 mm shunt.**
***Conclusions.*** **It is possible to achieve a partial portacaval shunt, related to the diameter of the prosthesis, that preserves hepatopetal flow in the majority of patients and is associated with a very low incidence of shunt thrombosis. This effectively prevents recurrent variceal bleeding and significant postoperative encephalopathy. The performance of subsequent orthotopic liver transplantation is not compromised. The technique is recommended, either as a primary procedure or when sclerotherapy has failed, in patients with good liver function who are unlikely to require early liver transplantation (grade A and some grade B cirrhosis). (SURGERY 1992; 111:610–6.)**

**From the Hepato-biliary Surgery and Liver Transplant Research Unit, South Paris Faculty of Medicine, Hopital Paul Brousse, Villejuif, France.**

KEY WORDS: Portacaval shunt, PTFE portacaval shunt, partial portacaval shunt

### PAPER DISCUSSION

This important paper by Bismuth's group, who introduced the concept of partial diversion of the portal system in 1967, provides further compelling support for the efficacy of small diameter interposition ringed polytetrafluoroethylene (PTFE) prosthetic portacaval shunts in patients with variceal haemorrhage. Operative mortality was very low (4%), shunt patency up to 4 years was 96%, there was no recurrence of variceal haemorrhage and no patients developed chronic encephalopathy. It is to be noted, however, that only 33% of these patients had alcoholic cirrhosis, that 68% were Child-Pugh grade A, 32% were grade B and none were grade C.

The results should be compared with those of Sarfeh *et al.*,[1] who pioneered the use of small diameter ringed PTFE shunts in 1983. In a systematic appraisal of diameters of PTFE interposition prostheses (8 to 16 mm) they found that optimal results in terms of maintained mesenteric venous pressure, prograde portal venous perfusion of the liver and incidence of postoperative encephalopathy were obtained with 8 mm diameter grafts. No patients with 16 to 20 mm diameter grafts, and only 1 of 12 with 12 to 14 mm diameter grafts retained prograde portal flow and the overall incidence of encephalopathy in these groups was 39%. With 10 mm diameter shunts prograde portal flow was maintained in 46% and the incidence of encephalopathy was 19%. With 8 mm diameter grafts prograde flow was present in over 80% of patients (9 of 11) and the incidence of encephalopathy was 9%. Late patency of grafts, up to 5 years, was 97% and no patients with 8 or 10 mm grafts rebled from varices.

In the present series the majority (19 of 25) patients had 10 mm diameter grafts, 4 patients had 12 mm diameter grafts and 8 mm grafts were used in only 2 pati- ents. Portal perfusion data were available only in 10 of the 19 patients with 10 mm grafts and in these prograde perfusion was demonstrable in 70%, compared with 46% in Sarfeh's study. If the findings in these 10 patients are representative of the whole subgroup of 19 patients with 10 mm grafts, and if Sarfeh's findings with 12 mm diameter grafts can be extrapolated to the 4 patients with 12 mm grafts in this series it is possible that 17 of the entire group of 25 patients (68%) lost prograde portal perfusion. This must be viewed in the context of the zero incidence of chronic encephalopathy in the entire series. This suggests that there is no straight correlation between the occurrence of encephalopathy and maintenance of prograde portal venous perfusion.

This is supported by the results of a recent study by Johansen[2] comparing total (2.5 cm) with partial (1.2 to 1.5 cm) side-to-side portacaval shunts. The encephalopathy incidence in partial (small diameter) shunts was 8% versus 56% with total portocaval shunts. There was also a significant late survival advantage in patients with partial shunts (13% vs 39% late deaths). Angiographic and Duplex sonographic studies revealed that *all* patients in both groups had reversal of flow in the distal limb of the portal vein, indicating uniform loss of first-pass hepatic portal perfusion. This suggests that besides hepatic portal perfusion, other factors must be operative in the genesis of hepatic and neurological dysfunction after shunting.

This is also relevant to the situation after distal splenorenal shunts (DSRS). Our[3] and other studies have documented the low incidence of encephalopathy after DSRS. This occurs despite the apparent loss of prograde portal venous perfusion with time in up to 50% of patients with alcoholic cirrhosis after DSRS reported by Warren *et al*[4]. In our study of 141 patients with DSRS[3,5] the frequency of failure to demonstrate prograde portal venous perfusion by DTPA hepatic flow scintigraphy only occurred in alcoholic cirrhotics, but was less (23%) than reported by Warren, and there was no difference in the incidence of encephalopathy between alcoholic cirrhotics and non-alcoholic *cirrhotics*. The incidence of encephalopathy was zero in *non-cirrhotic* patients after DSRS (mainly schistosomiasis and portal vein thrombosis).

The continuing debate about the apparent loss of selectivity after DSRS is therefore of questionable relevance in the light of the above, and the clinical[2] and experimental[6] evidence for the importance of preservation of physiologic mesenteric venous pressures in the prevention of postshunt hepatic functional deterioration and encephalopathy. It is suggested that this may well be largely responsible for the excellent results in this regard following DSRS and partial portacaval decompression with small diameter PTFE interposition grafts.

The confirmation of Sarfeh's experience of durable patency with the use of small diameter ringed PTFE interposition prosthetic grafts appears to have allayed residual reservations about this procedure. Further experience over more extended periods will be required to establish whether it will truly match the excellent long term patency rate and functional results of the DSRS. In my view it has established a place in the operative therapeutic armamentarium for portal hypertension, especially for patients where a DSRS is impossible, for example patients who have undergone a previous ill advised splenectomy. The technical simplicity and safety of this procedure are also appealing in the less common context of emergency shunting, although it is doubtful whether it will have a major impact on the much greater operative mortality experienced with all forms of emergency operative intervention in Childs grade C patients.

*References*

1. Sarfeh, I. J., Rypins, E. B. and Mason, G. R. (1986) A systematic appraisal of portacaval H-graft diameters. *Ann. Surg.*, **204**, 356–63.
2. Johansen, K. (1992) Prospective comparison of partial versus total portal decompression for bleeding esophageal varices. *Surg. Gynecol. Obstel.*, **175**, 528–34.
3. Myburgh, J. A. (1990) Selective shunts: the Johannesburg experiences. *Am. J. Surg.*, **160**, 67–72.
4. Warren, W. D., Millikan, W. J. and Henderson, J. M. *et al.* (1984) Selective variceal decompression after splenectomy or splenic vein thrombosis with a note on splenopancreatic disconnection. *Ann. Surg.*, **199**, 694–72.
5. Myburgh, J. A. (1991) The Warren shunt: Effect of alocoholism on portal perfusion. Discussion on paper by Kawasaki, S. *et al. HPB Surgery*, **5**, 70–73.
6. Johansen, K. H., Girod, C., Lee, S. S. and Lebrec, D. (1990) Mesenteric venous stenosis reduces hyper-ammonemia in the portacaval shunted rat. *Eur. Surg. Res.*, **22**, 170–74.

J. A. Myburgh
Professor of Surgery
University of the Witwatersrand
Medical School
York Road
Parktown, Johannesburg
2193, South Africa

### GASTRIC VARICES: FIRST YOU HAVE TO SEE THEM

### ABSTRACT

*Sarin, S. K., Lahoti, D., Saxena, S. P., Murthy, N. S., and Makwana, U. K. (1992) Prevalence, Classification and Natural History of Gastric Varices: A longterm follow-up study in 568 portal hypertension patients. Hepatology, 16, 1343–1349.*

**To determine the prevalence and natural history of gastric varices, we prospectively studied 568 patients (393 bleeders and 175 nonbleeders) with portal hypertension (cirrhosis in 301 patients, noncirrhotic portal fibrosis in 115 patients, extrahepatic portal vein obstruction in 117 patients and hepatic venous outflow obstruction in 35 patients). Primary (present at initial examination) gastric varices were seen in 114 (20%) patients; more were present in bleeders than in nonbleeders (27% vs. 4%, respectively; $p < 0.001$). Secondary (occurring after obliteration of esophageal varices) gastric varices developed in 33 (9%) patients during follow-up of $24.6 \pm 5.3$ mo. Gastric varices (compared with esophageal varices) bled in significantly fewer patients (25% vs. 64%, respectively). Gastric varices had a lower bleeding risk factor than did esophageal varices ($2.0 \pm 0.5$ vs. $4.3 \pm 0.4$, respectively) but bled more severely ($4.8 \pm 0.6$ vs. $2.9 \pm 0.3$ transfusion units per patient, respectively). Once a varix bled, mortality was more likely (45%) in gastric varix**

